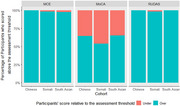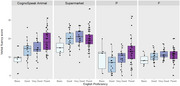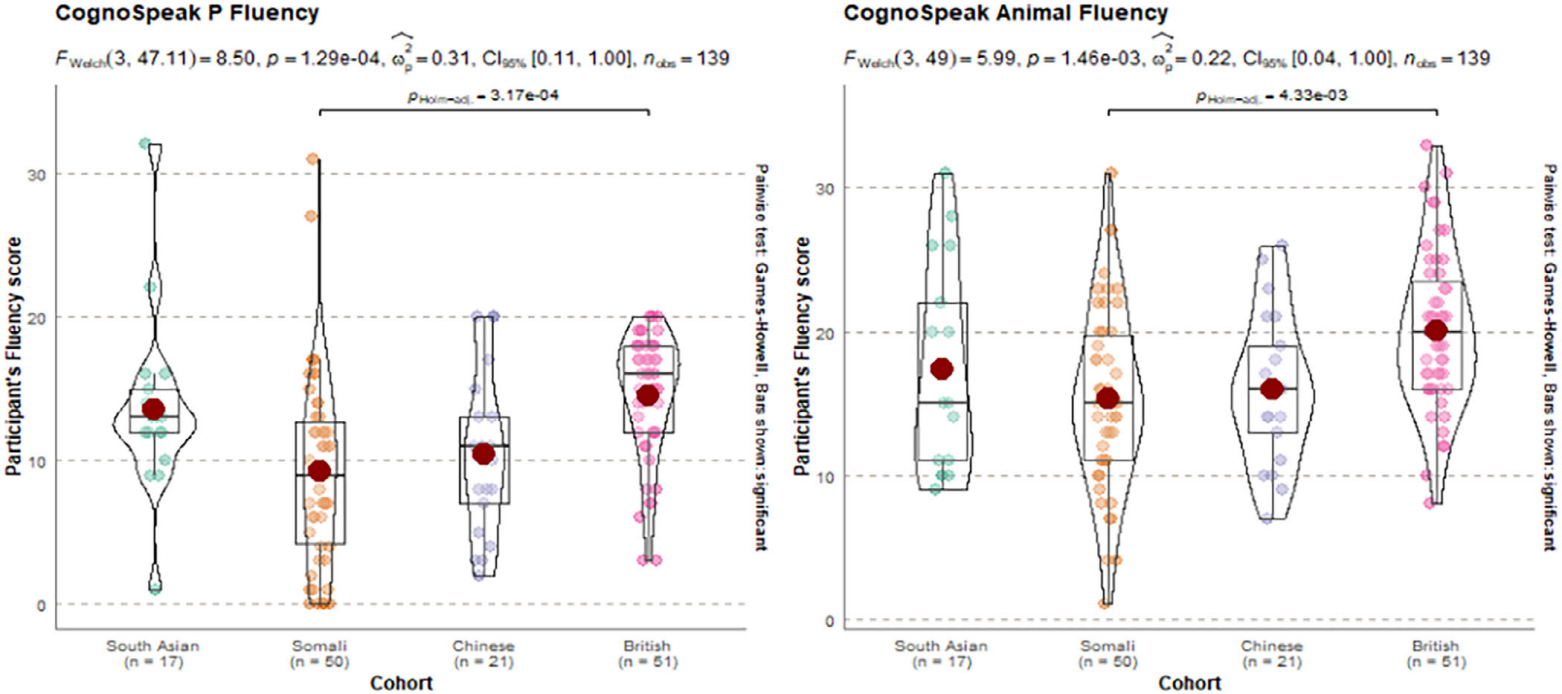# Developing a more equitable language‐based automated assessment of cognition: CognoSpeak

**DOI:** 10.1002/alz70857_096485

**Published:** 2025-12-24

**Authors:** Caitlin H Illingworth, Hina Khan, Dorota Anna Braun, Lise Sproson, Madhurananda Pahar, Bahman Mirheidari, Heidi Christensen, Daniel J. Blackburn, Sarah Goodwin

**Affiliations:** ^1^ University of Sheffield, Sheffield, South Yorkshire, United Kingdom; ^2^ NIHR Health Research Centre in Long Term Conditions ‐ Devices for Dignity for Dignity, Sheffield, United Kingdom

## Abstract

**Background:**

Current common cognitive assessment tools more frequently misdiagnose patients from minority ethnic groups and those who speak English as an additional language. CognoSpeak is a language‐based memory assessment tool underpinned by artificial intelligence. Avoiding bias within this system requires exposure to a diverse training population.

**Methods:**

Research champions from within South Asian, Somali, and Chinese community groups were trained to recruit and assess participants using different pen‐and‐paper (Rowland Universal Dementia Assessment Scale (RUDAS), Multicultural Cognitive Examination (MCE), Montreal Cognitive Assessment (MoCA)) and automated (CognoSpeak) cognitive assessments.

**Results:**

To date, 146 participants (52 Somali, 53 South Asian, and 41 Chinese) have been recruited through these community centres, making up over 60% of the total number of BAME participants in CognoSpeak's training cohort (*n* >1500). Despite all participants being cognitively intact, preliminary data shows that the MoCA miscategorised 39.8% of the participants as cognitively impaired, compared to just 0.8% on the RUDAS and the MCE (*p<*.001). Initial analysis indicates that Somali participants scored significantly lower than monolingual English speakers across both CognoSpeak's verbal fluency tasks (Semantic: Animals, phonemic: ’P’) (*p<*.001). However, Somali does not have the phoneme “P” meaning the fluency score may be artificially low, reflecting the importance of considering language background in cognitive assessments. We will present the relative accuracy of CognoSpeak's categorisation of participants from these cohorts compared to a White English Speaking monolingual cohort.

**Conclusion:**

Despite common usage, the MoCA may not be appropriate for minoritised communities and individuals who speak English as an additional language. This collaborative approach helped break down traditional barriers to research participation. We will expand these results to present the accuracy of CognoSpeak across different language backgrounds and ethnicities.